# The Attentional Boost Effect in Young and Adult Euthymic Bipolar Patients and Healthy Controls

**DOI:** 10.3390/jpm11030185

**Published:** 2021-03-06

**Authors:** Giulia Bechi Gabrielli, Clelia Rossi-Arnaud, Pietro Spataro, Fabrizio Doricchi, Marco Costanzi, Alessandro Santirocchi, Gloria Angeletti, Gabriele Sani, Vincenzo Cestari

**Affiliations:** 1 Department of Psychology, Faculty of Medicine and Psychology, Sapienza University, 00185 Rome, Italy; giulia.bechigabrielli@uniroma1.it (G.B.G.); clelia.rossi-arnaud@uniroma1.it (C.R.-A.); fabrizio.doricchi@uniroma1.it (F.D.); alessandro.santirocchi@uniroma1.it (A.S.); 2 Faculty of Economics, Universitas Mercatorum, 00100 Rome, Italy; pietro.spataro@unimercatorum.it; 3 Department of Human Sciences, Lumsa University, 00100 Rome, Italy; m.costanzi@lumsa.it; 4 Nesmos Department, Sapienza University, Sant’Andrea Hospital, 00100 Rome, Italy; gloria.angeletti@uniroma1.it; 5 Institute of Psychiatry, Università Cattolica del Sacro Cuore, 00100 Rome, Italy; gabriele.sani@unicatt.it; 6 Department of Psychiatry, Fondazione Policlinico Universitario “Agostino Gemelli” IRCCS, 00100 Rome, Italy

**Keywords:** Attentional Boost Effect, bipolar disorder, euthymic patients, recognition memory

## Abstract

In the Attentional Boost Effect (ABE), stimuli encoded with to-be-responded targets are later recognized more accurately than stimuli encoded with to-be-ignored distractors. While this effect is robust in young adults, evidence regarding healthy older adults and clinical populations is sparse. The present study investigated whether a significant ABE is present in bipolar patients (BP), who, even in the euthymic phase, suffer from attentional deficits, and whether the effect is modulated by age. Young and adult euthymic BP and healthy controls (HC) presented with a sequence of pictures paired with target or distractor squares were asked to pay attention to the pictures and press the spacebar when a target square appeared. After a 15-min interval, their memory of the pictures was tested in a recognition task. The performance in the detection task was lower in BP than in HC, in both age groups. More importantly, neither young nor adult BP exhibited a significant ABE; for HC, a robust ABE was only found in young participants. The results suggest that the increase in the attentional demands of the detection task in BP and in adult HC draws resources away from the encoding of target-associated stimuli, resulting in elimination of the ABE. Clinical implications are discussed.

## 1. Introduction

The Attentional Boost Effect (ABE) represents a counterintuitive phenomenon in which the division of attention at encoding enhances later memory performance [1,2,3] (see Swallow and Jiang [4] for a review). In the latest version of the paradigm [5], participants were presented with a series of faces flanked by two target squares (e.g., orange), two distractor squares (e.g., blue), or no squares (the baseline condition). Participants were required to study the faces and simultaneously press the spacebar when the target squares appeared. When their memory of the faces was later probed in a yes/no recognition task, the performance was significantly better for the faces which were presented with target squares than for those presented with distractor squares or no square at all; no difference in performance was found between the latter two. This advantage of target-paired over distractor-paired images is referred to as the ABE. It is thought to be the result of a broad attentional enhancement linked to the detection of target squares [2,5]. More specifically, in their dual-task interaction model, Swallow and Jiang [4] proposed that, on the one hand, monitoring the squares interferes with image encoding, because it biases perceptual resources away from the background stimuli and places additional cognitive demands to generate an appropriate response [6]. On the other hand, detecting the target squares and performing the associated motor response triggers temporal selective attention [7]. This mechanism facilitates perceptual processing of the background images by producing a transient increase in the release of norepinephrine from the locus coeruleus [8] (see Yebra et al. [9] for recent evidence). Under specific conditions, this processing enhancement exceeds the usual interference effects, leading to the ABE.

Despite being a recently described phenomenon, the ABE has been extensively investigated. First, it has been replicated with verbal materials in a variety of explicit memory tasks, including yes/no and forced-choice recognition [2,10,11], cued recall [12], and free recall [10]; the ABE has also been reported in perceptual implicit [3] and working memory tasks [13,14]. Second, studies have ruled out several potential explanations of the ABE, including accounts based on perceptual learning, target distinctiveness, attentional cueing, reinforcement learning, and perceptual grouping [2,15,16]. Third, studies that have included separate full-attention (FA) and divided-attention (DA) conditions have shown that the ABE represents a dynamic trade-off between attentional competition and attentional facilitation [2,3,4,10,17]. Here, the term ‘attentional competition’ refers to the finding that recognition of distractor-paired items is usually worse in the DA condition (in which participants have to encode the background stimuli and simultaneously detect the targets) than in the FA condition (in which the sole task is to encode the background stimuli), confirming the classical negative effects of DA on memory encoding. In contrast, the term ‘attentional facilitation’ refers to the finding that the recognition of target-paired items in the DA condition is boosted to the same level of the FA [2,10,17]. The ABE reflects a condition in which attentional facilitation exceeds attentional competition.

Most of the above-summarized evidence was gathered in studies of healthy young participants, typically university students. To date, only a handful of studies have examined the ABE in clinical populations [18,19,20,21]. Furthermore, except for a study by Rossi-Arnaud et al. [21], these studies used a modified version of the original ABE paradigm similar to the Rapid Serial Visual Presentation (RSVP) [13,14,22], in which participants encoded brief sequences of stimuli and the recognition task was administered immediately after the end of each sequence. Collectively, the results of these studies showed that patients with both amnestic mild cognitive impairment and post-traumatic stress disorder were severely impaired in terms of the recognition of scenes that were paired with targets. In contrast, these same patients performed like controls, or even displayed an enhanced performance, in terms of the recognition of scenes that were paired with distractors [19,20]. A different pattern was obtained in patients with Parkinson’s disease before and after the administration of dopaminergic medications. At the baseline, drug-naive patients performed like controls. However, after receiving dopamine agonists for 12 weeks, patients outperformed controls for both target-paired and distractor-paired stimuli [18]. Only one previous study [21] has investigated the ABE in patients with schizophrenia, using a paradigm similar to that illustrated by Swallow and Jiang [2,5], in which patients and healthy controls encoded a long series of stimuli and the recognition test was separated from the encoding phase. The results showed that, unlike controls who displayed the typical ABE, patients with schizophrenia exhibited no memory advantage for target-paired pictures (Exp. 1) and words (Exp. 2).

The present study focused on bipolar disorder, which is characterized by mood alterations that include manic or hypomanic episodes (in which there is an elevation of tone mood), depressive episodes (in which there is a decline of tone mood), and mixed episodes, intermingled with intervals of euthymic remission [23]. We chose to examine this clinical population because there is growing evidence that individuals with bipolar disorder are affected by significant attentional deficits [24,25,26,27,28], even after controlling for mild residual symptomatology [29] and pharmacological treatment [30,31]. In particular, in the remission phase, bipolar patients (euthymic patients) show a decreased target sensitivity (omission errors) and slowed response latencies in detection tasks that require sustained attention [29,32,33,34,35,36,37,38], in which participants have to monitor a continuous stream of stimuli to detect a pre-specified target.

Based on these results, our primary aim was to investigate the ABE in a sample of euthymic patients, using the most recent version of the paradigm [5]. Following the notion mentioned above, that the ABE represents a trade-off between attentional competition and attentional facilitation [2,3,4,10,17], we expected euthymic patients to exhibit a reduced or non-significant advantage for images encoded with target stimuli. Swallow and Jiang [2] (Exp. 5) showed that, when target detection requires additional attention resources, the negative effects of attentional competition exceeded the positive effects of attentional facilitation, thus resulting in elimination of the ABE. We reasoned that, if the maintenance of a fast and accurate performance in the target detection task requires more attention resources in euthymic patients than in healthy controls (as suggested by previous studies [29,32,33,35,36,37]), then the ABE should have been reduced or eliminated in the patient group.

In addition to testing the ABE in euthymic patients, a secondary aim of the present study was to investigate whether participants’ age modulates this effect. Previous studies have typically reported a strong ABE in young adults between 18 and 35 years of age. In contrast, relatively few data have been reported for older participants and the overall findings are mixed. Bechi Gabrielli, Spataro, Pezzuti and Rossi-Arnaud [39] found that the ABE was abolished in older adults between 60 and 75 years when a 20-min interval separated the encoding and test phases. On the other hand, Prull [17], with a short study-test interval (2 min), reported that young-old adults (between 60 and 75 years) exhibited an ABE with a magnitude that did not differ from that of young adults, although a significant decrease was observable in older-old adults (from 75 to 94 years). Based on these results, Prull [17] speculated that the cognitive decline associated with healthy aging might produce vulnerable boosted memories that would be more easily disrupted over time, compared to the boosted memories of young adults; that is, due to processing resource limitations and/or associative deficits, long study-test delays would create a selective interference that impairs the boosted memories of older adults. The putative mechanisms underlying this impairment have been examined by a series of neuroimaging studies investigating the cerebral bases of the ABE. According to Swallow and Jiang [4], target detection in the ABE paradigm results in a transient increase in the release of norepinephrine from the locus coeruleus (LC), which in turn projects to a wide variety of cortical regions, including the hippocampus. More recent studies have showed that the memory enhancements following ABE manipulations are associated with widespread increases in functional connectivity between the LC and the parahippocampal gyrus [9] and between the visual cortex and the hippocampal formation [40]. Interestingly, for the present purposes, healthy aging is accompanied by (a) increased tau pathology and a reduced density of the LC, which are already evident from 20 years onwards [41], and (b) substantial shrinkage of the regional brain volume of the hippocampus, which accelerates with age (from 20 to 90 years) [42]. In addition to accounting for the creation of vulnerable boosted memories in older adults, the age-related changes occurring in these two structures suggest an additional inference: Namely, that significant reductions in the size of the ABE should be apparent in healthy adults well before the age of 60 years, at least when using long study-test intervals. To address this issue, in the present study, both the patient and healthy control samples were divided into two sub-groups: The young group included participants between 18 and 35 years, whereas the adult group included participants between 36 and 60 years. This allowed us to examine, for the first time, whether there is a significant reduction in the size of the ABE in the adult group. 

## 2. Materials and Methods

Forty-two euthymic bipolar patients (BP) Type I, between 18 and 60 years, were recruited for the current study from the Psychiatric Ward of the Sant’Andrea Hospital in Rome. As stated above, they were divided into a young subgroup (*n* = 12; 5 females; age: M = 27.4 years, range: 18–35 years; education: M = 13.7) and an adult subgroup (*n* = 30; 17 females; age: M = 50.3 years, range: 36–61 years; education: M = 12.7). The diagnosis of bipolar disorder was made according to the inclusion criteria specified by the DSM-5 [23]. All patients were under pharmacological treatment at the time of the study: Specifically, 33 patients (79%) were administered antipsychotics (e.g., seroquel, zyprexa, leponex, etc.); 36 (88%) were administered mood stabilizers (e.g., carbolithium, depakin, etc.); 14 (34%) were administered anxiolytics (e.g., diapezam); and 7 (16%) were administered antidepressants (e.g., anafranil, zoloft, etc.). To be included, patients had to be in the euthymic phase [23]. Forty-two healthy control subjects (HC), from 18 to 60 years old, were recruited as controls. They were likewise divided into a young subgroup (*n* = 15; 6 females; age: M = 26.7 years, range: 22–35 years; education: M = 12.7) and an adult subgroup (*n* = 27; 16 females; age: M = 51.1 years, range: 37–60 years; education: M = 13.5). Eight participants (3 from the BP group and 5 from the HC group) were additionally tested but excluded from statistical analyses because their accuracy in the detection task or the memory test fell two or more standard deviations below the overall mean. Four subtests of the WAIS-IV [43,44] were administered to all participants—the Digit Span subtest (forward and backward) to evaluate the working memory, and the Symbol Search and Digit Symbol-Coding subtests to evaluate the processing speed.

Potential differences in demographic characteristics and cognitive scores between bipolar patients and healthy controls were analyzed through a series of *t*-tests for independent samples. Separate analyses were conducted in the two age subgroups (see Table 1). In the young subgroup, significant differences were only found in the Symbol Search subtest of WAIS-IV, indicating lower scores in bipolar patients than in healthy controls: t(25) = 2.13, and *p* = 0.042. For the adult subgroup, significant differences were similarly obtained in the speed subtests of WAIS-IV, again indicating lower scores in bipolar patients than in healthy controls: t(55) = 3.04 and *p* = 0.004 for Symbol Search and t(55) = 3.02 and *p* = 0.004 for Digit Symbol-Coding. Bipolar patients and healthy controls were matched in terms of age and gender, as well as in the distribution of gender. 

The study was carried out at the Sant’Andrea Hospital in compliance with ethical guidelines and written informed consent was obtained from each participant. Both healthy controls and bipolar patients participated in the study voluntarily.

A critical set of 45 neutral pictures were selected from the International Affective Picture System (IAPS) [45] valence: M = 5.28, on a 9-point Likert scale ranging from 1 = unhappy to 9 = very happy; arousal: M = 3.18, on a 9-point Likert scale ranging from 1 = relaxed to 9 = excited) (see Rossi-Arnaud, Spataro, Costanzi, Saraulli, and Cestari [1] for a study examining the ABE with emotional words and images). This initial set was further divided into three subgroups of 15 images. Each image could be associated with a red square (target condition); associated with a green square (distractor condition); or presented on its own, without squares (baseline condition). The use of the three subsets of images in the different encoding conditions was counterbalanced across participants. An additional set of 124 non-critical neutral images were also selected from the IAPS, to be used as practice (5 images) and filler items (74 images) during the encoding phase, or as foils in the recognition task (45 images). Foils were as similar as possible to the critical images in terms of valence (M = 5.29) and arousal (M = 3.17). All images were pre-processed with Adobe Illustrator CS6 and presented on the 15” monitor of an HP Pavilion notebook using the software SuperLab 4.0 (Cedrus Corporation, San Pedro, CA, USA). 

The experiment comprised an encoding phase, a 15-min interval, and a test phase (Bechi Gabrielli et al., 2018, Exp.1). In the encoding phase, participants were presented with a total of 124 images, at a rate of 500 ms/picture (no inter-stimulus interval). All of the stimuli were displayed on the 15˝ display of an HP Pavilion notebook, with participants being sat at a distance of about 40 cm. For target and distractor trials, one image (1024 × 628 pixels) and one square (70 × 70 pixels; red or green, placed at the center of the image) appeared simultaneously on the screen for 100 ms, after which only the image remained visible for an additional 400 ms. For baseline trials, the images were presented for 500 ms, without squares. The entire presentation was divided into 16 continuous blocks of five images each (one practice block plus 15 critical blocks). Each block included 1 target image (presented with a red square), 1 distractor image (presented with a green square), 1 baseline image (presented without squares), and 2 filler images (presented with green squares). The target image was always located in the third position, whereas the distractor and baseline images were located in either the first or fifth position (the exact position was counterbalanced across blocks). In addition, from one to five filler images, always presented with green squares, were placed between adjacent blocks to reduce the regularity in the appearance of the target squares. Participants were told to pay attention to the images (incidental instructions, since they were not forewarned about the impending memory task) and simultaneously press the spacebar whenever they detected a red square. During the 15-min interval, both healthy controls and bipolar patients undertook the four WAIS-IV subtests. Finally, the recognition task involved the random presentation of 90 images, 45 old images (presented at encoding, including 15 target-paired, 15 distractor-paired, and 15 baseline images) and 45 new images (foils). For each image, the instructions were to press the key “v” (for “vecchio”, old) or “n” (for “nuovo”, new) if the participant judged it to be old or new, respectively. 

## 3. Results

At encoding, the performance in the detection task was analyzed via a 2 × 2 ANOVA, considering group (healthy controls, HC vs. bipolar patients, BP) and age (young, Y vs. adult, A participants) as between-subject factors. The dependent variables were the mean percentages of targets correctly detected, the mean numbers of false alarms to distractor or baseline trials, and the mean detection times. The results showed that bipolar patients and healthy controls were equally accurate in the detection of target squares (M(HC) = 93.4% vs. M(BP) = 89.7%, F(1,80) = 2.90, *p* = 0.09, and η2 = 0.04). Bipolar patients made more false alarms than healthy controls, although the overall percentages were very low (M(HC) = 0.23% vs. M(BP) = 1.00%, F(1,80) = 7.4, *p* = 0.008, and η2 = 0.09). Finally, both groups were equally faster in target detection (M(HC) = 344.1 ms vs. M(BP) = 329.7 ms, F(1,80) = 1.95, *p* = 0.17, and η2 = 0.02). When we analysed the main effects of age, we found that young and adult participants were equally accurate in the detection task (M(Y) = 92.5% vs. M(A) = 90.7%, F(1,80) = 0.69, *p* = 0.41, and η2 = 0.01), and the two groups did not differ in the mean percentages of false alarms (M(Y) = 0.47% vs. M(A) = 0.75%, F(1,80) = 0.95, *p* = 0.33, and η2 = 0.01). They were also equally faster in the detection of target squares (M(Y) = 337.1 ms vs. M(A) = 336.6 ms, F(1,80) = 0.002, *p* = 0.96, and η2 = 0.00). We did not find significant interactions in any analysis.

For the recognition test, we first analysed the proportions of false alarms with a 2 (group: healthy controls vs. bipolar patients) × 2 (age: young vs. adult participants) ANOVA. We did not find a main effect of group: The mean proportions of false alarms were comparable between bipolar patients and healthy controls (M(HC) = 0.15 vs. M(BP) = 0.18, F(1,80) = 1.23, *p* = 0.27, and η2 = 0.02). On the contrary, we found a significant main effect of age (F(1, 80) = 6.71, *p* = 0.01, and η2 = 0.08), indicating that adult participants (M = 0.20) committed more false alarms than young participants (M = 0.13). For this reason, all the subsequent statistical analyses were conducted on corrected recognition scores, computed as hits minus false alarms (this is a common procedure in studies examining the ABE in recognition tasks [1,21,39]). Note that the proportions of hits were adjusted by only considering those trials in which participants correctly performed the detection task [10]. These adjusted scores were submitted to a 2 (group: healthy controls vs. bipolar patients) × 2 (age: young vs. adult participants) × 3 (type of trial: target, distractor, and baseline images) mixed ANOVA. The results showed (a) a marginal main effect of trial type (F(2, 160) = 2.96, *p* = 0.054, and η2 = 0.04): The post-hoc comparisons demonstrated that the recognition of distractor-paired images (M = 0.26) was significantly worse than the recognition of baseline images (M = 0.32, *p* = 0.03); (b) a significant main effect of group (F(1,80) = 7.40, *p* = 0.008, and η2 = 0.09), indicating that healthy controls (M = 0.35) performed the recognition task significantly better than bipolar patients (M = 0.25); and (c) a significant two-way interaction between group and trial type (F(2, 160) = 4.59, *p* = 0.01, and η2 = 0.05), and a significant three-way interaction between group, age, and trial type (F(2, 160) = 4.92, *p* = 0.008, and η2 = 0.06). All other effects and interactions failed to reach the significance level (all Fs (1,80) < 2.01, *p* > 0.16). 

**Figure 1:**
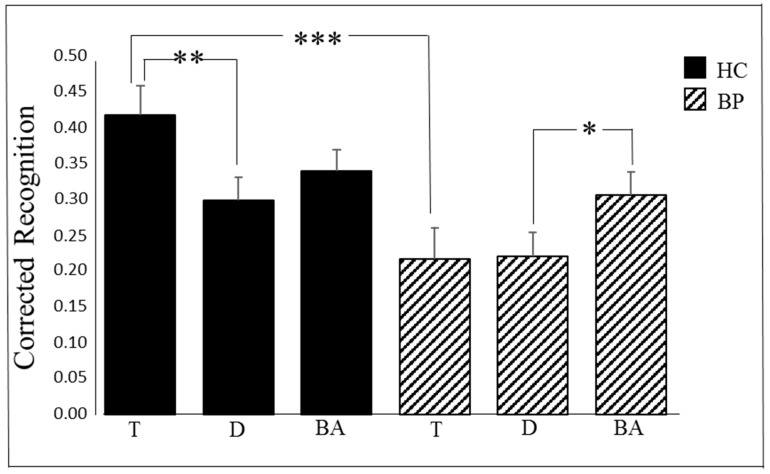
Mean proportions of corrected recognition (hits—false alarms) in bipolar patients (BP) and healthy control subjects (HC) as a function of trial type. Bars represent *SE*s. **Note**: * *p* < 0.05; ** *p* < 0.01; *** *p* < 0.001; T, target images; D, distractor images; BA, baseline images; HC, healthy controls; and BP, bipolar patients.

A follow-up analysis of simple effects on the two-way interaction between group and trial type (see Figure 1) revealed that the effect of trial type was significant in healthy controls (F(2, 79) = 4.28, *p* = 0.017, and η2 = 0.10). For this group, the recognition of target-paired images (M = 0.42) was significantly more accurate than the recognition of distractor-paired images (M = 0.30, *p* = 0.013)—the Attentional Boost Effect. On the contrary, the recognition of baseline images (M = 0.34) did not differ from the recognition of target and distractor-paired images (*p* = 0.23 and *p* = 0.67, respectively). The effect of trial type was also significant in bipolar patients (F(2, 79) = 3.36, *p* = 0.040, and η2 = 0.08): For this group, the recognition of baseline images (M = 0.31) was significantly better than the recognition of distractor-paired images (M = 0.22, *p* = 0.048), but did not differ from the recognition of target-paired images (*p* = 0.18); no differences were found between the recognition of target-paired and distractor-paired images (*p* = 1.00).

When analysed in the opposite direction, this same interaction indicated that the effect of group was significant for target-paired images, with healthy controls (M = 0.42) outperforming bipolar patients (M = 0.22) (F(1, 80) = 11.31, *p* = 0.001, and η2 = 0.12). The two groups did not differ in the recognition of distractor-paired and baseline images (F(1, 80) = 2.87, MSE = 0.038, *p* = 0.094, and η2 = 0.04 and F(1, 80) = 0.54, *p* = 0.46, and η2 = 0.01, respectively).

**Figure 2:**
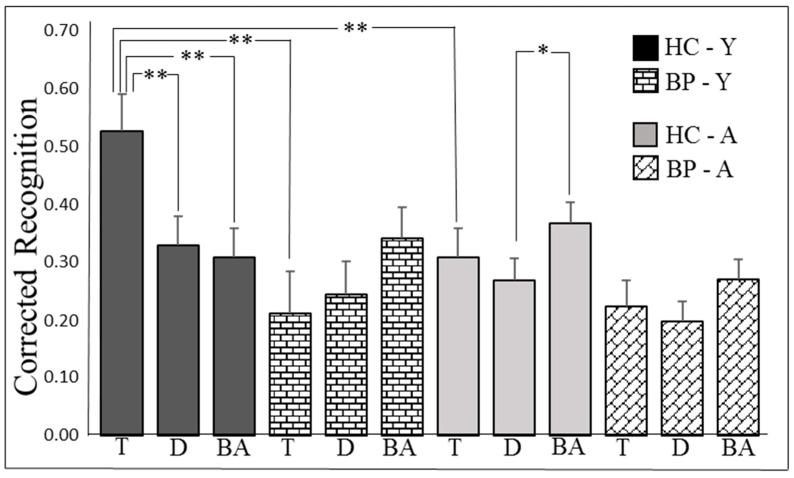
Mean proportions of corrected recognition (hits—false alarms) in bipolar patients (BP) and healthy control subjects (HC) as a function of trial type and age range (Y—young-adults, A—adult subjects). Bars represent *SE*s. **Note**: * *p* < 0.05; ** *p* < 0.01; T, target images; D, distractor images; and BA, baseline images.

A similar follow-up analysis of simple effects on the three-way interaction between group, age, and trial type (see Figure 2) revealed that the effect of trial type was significant for both young and adult healthy controls (F(2, 79) = 5.45, *p* = 0.006, and η2 = 0.12, and F(2, 79) = 3.19, *p* = 0.047, and η2 = 0.07, respectively). For young healthy controls, the recognition of target-paired images (M = 0.53) was significantly higher than the recognition of distractor-paired (M = 0.33, *p* = 0.01) and baseline images (M = 0.31, *p* = 0.008); no differences were found between these two conditions (*p* = 1.0). In contrast, adult healthy controls recognized baseline images (M = 0.37) better than distractor-paired images (M = 0.27, *p* = 0.039); the recognition of target-paired images (M = 0.31) did not differ from the recognition of distractor-paired and baseline images (*p* = 0.10 and *p* = 0.78, respectively). The effect of trial type was not significant in young and adult bipolar patients (F(2, 79) = 1.84, *p* = 0.17, and η2 = 0.04 and F(2, 79) = 1.97, *p* = 0.15, and η2 = 0.05, respectively), indicating no between-trial differences in these two subgroups. The same analysis showed that the effect of group was significant for young participants in the target condition (F(1, 80) = 10.25, MSE = 0.064, *p* = 0.002, and η2 = 0.11), and marginally significant for adult participants in the baseline condition (F(1, 80) = 3.72, *p* = 0.057, and η2 = 0.04). Therefore, young healthy controls recognized target-paired images (M = 0.53) more accurately than young bipolar patients (M = 0.21); similarly, adult healthy controls (M = 0.37) recognized baseline images more accurately than adult patients (M = 0.27). The effect of group failed to reach the significance level in all other conditions (all Fs(1, 80) < 1.88, *p* > 0.17). Lastly, the follow-up analysis indicated that the effect of age was significant for healthy controls in the target condition (F(1, 80) = 7.07, *p* = 0.009, and η2 = 0.08), indicating that young healthy controls recognized target-paired images (M = 0.53) more accurately than adult healthy controls (M = 0.31). The effect of age failed to reach the significance level in all other conditions (all Fs(1, 80) < 1.16, *p* > 0.28).

## 4. Discussion

In the present study, using the most recent version of the paradigm by Swallow and Jiang [5], we examined the ABE in a sample of young (18–35 years) and adult (36–60 years) euthymic bipolar patients and in samples of matched healthy controls. The results showed that, during the encoding phase, bipolar patients were as accurate and fast as healthy controls in detecting the target squares, but produced significantly more false alarms. However, the overall incidence of false alarms was low in both groups. Turning to the recognition task, young healthy controls showed the typical ABE, with target-paired images being recognized better than distractor-paired images. In contrast, the ABE was abolished in adult healthy controls and bipolar patients, irrespective of age; in the latter group, the recognition of baseline images was significantly higher than the recognition of distractor images, suggesting enhanced attentional competition. Finally, healthy controls outperformed bipolar patients in the recognition of target images, whereas the two groups were equally accurate in the recognition of distractor and baseline images. 

As mentioned in the introduction, the ABE represents a trade-off between attentional competition and attentional facilitation [2,4], such that any increase in the attentional requirements of the detection task should impair the encoding of target-associated stimuli, and thus reduce or even eliminate the memory facilitation produced. In agreement, Swallow and Jiang [2] (Exp. 5) showed that enhancing the difficulty of the detection task by asking participants to make different responses to target and distractor stimuli was sufficient to cancel the ABE. Several previous studies have already documented the attentional difficulties experienced by bipolar patients. They have shown that these deficits are not limited to the depressive and manic episodes, but extend to the euthymic phase [29,34,35,38]. Importantly, these patients show evident impairment in continuous performance tasks, in which they have to monitor a stream of stimuli to detect an infrequent pre-specified target [36,46]. Based on this literature, we expected that the maintenance of an accurate and fast performance in the detection task should recruit more attention resources in bipolar patients than in healthy controls and that the ensuing increase in the negative effect of attentional competition should eliminate the ABE. 

Two results from the present study provide support for this prediction. First, bipolar patients exhibited a selective impairment in the recognition of target-paired images, together with an intact performance in the detection of target squares. Since, in our study, participants were not explicitly required to remember the background stimuli and were unaware of the following recognition test, it can be plausibly assumed that both bipolar patients and healthy controls emphasized and devoted more attention resources to the detection task than to the memory task (see Bechi Gabrielli et al. [39] for a discussion). Our data suggest that the maintenance of a fast and accurate performance in the detection task required more attention resources in bipolar patients than in healthy controls. As a consequence, bipolar patients had fewer resources available to encode the target-paired images into memory, resulting in a significant and selective deficit in the recognition of these images. 

The second piece of evidence that supports this interpretation of the bipolar patients’ performance comes from the significant two-way interaction between group and type of trial. This interaction highlighted that bipolar patients recognized baseline images significantly more accurately than distractor-paired images. Previous studies that have compared the FA and DA conditions have pointed out that the recognition of distractors is significantly lower in the DA than in the FA condition. These findings reflect the classical negative effects of divided attention [2,10,17]. In the paradigm used in the present study, the difference between the recognition of distractor and baseline images has been similarly proposed to reflect the attentional competition component of the ABE [5]. In line with this idea, a recent study using the Remember/Know procedure found that the proportions of ‘remember’ responses were significantly lower for distractor-paired than baseline words [47]. If this were the case, the present results might suggest that the negative effects of DA were stronger in bipolar patients than in healthy controls. Since the ABE emerges from the interaction between attentional competition and attentional boost [2,4,10], the direct consequence of an increase in the interfering effects of attentional competition must necessarily be a reduction in the positive effects of the ABE. In sum, taken together with the selective impairment in the recognition of target-paired images, the finding mentioned above supports the idea that the maintenance of an adequate performance in the detection task is more attention-demanding in bipolar patients than in healthy controls and that the ensuing enhancement of the negative effects of DA was sufficient to eliminate the ABE. 

In our experiment, we also investigated whether participants’ age influenced the ABE. The large majority of previous studies have examined the ABE in young university students between 18 and 35 years [2,5,10,15]. To the best of our knowledge, only two studies were explicitly aimed at comparing the ABE of younger and older participants [17,39]. The results were mixed, likely because different study-test intervals (2 min vs. 20 min) were used in the two studies. On the basis of this evidence, Prull [17] proposed the so-called *vulnerable boost hypothesis*. Put simply, this hypothesis assumes that (a) maintaining boosted memories across a long study-test interval implies a substantial amount of interference, and (b) the negative effect of this interference would be larger in older than in younger adults, because of the reduced cognitive resources and/or the associative deficits commonly associated with aging. The results from two previous studies support this proposal. An advantage of target-paired images in older adult controls (age: M = 63.2 and M = 63.8 years) was found when using a short-term version of the ABE paradigm [18,20]. These results should be taken together with Prull’s observation [17] of a significantly reduced size of the ABE in older-old adults, even when a very short 2-min study-test interval was used. Overall, these results suggest that the negative effects of interference increase linearly with age, such that (a) young adults show the ABE after both a short and long study-test interval; (b) young-old adults show the ABE after a short interval, but the effect is reduced or eliminated after a long interval; and (c) older-old adults already show a reduced ABE or no effect after a short interval.

To further clarify this issue, we recruited healthy controls and bipolar patients ranging from 18 to 60 years. We divided both samples into two age-subgroups: A ‘young-adult’ group, from 18 to 35 years of age, and an ‘adult’ group, ranging in age from 36 to 60 years. In line with our expectations, such a division had a strong impact on the ABE, as demonstrated by the significant three-way interaction between group, age, and type of trial. The follow-up analyses confirmed that the ABE was significant in young-adult healthy controls: Replicating previous results, the images encoded with targets were recognized significantly better than the images encoded with distractors or presented without squares [1,2,3,5,10,11,39,48]. In contrast, the ABE was abolished in adult healthy controls. Most importantly, we also found that the mechanisms accounting for the elimination of the ABE were similar to those discussed previously for bipolar patients: The follow-up analyses of the three-way interaction indicated that adult healthy controls recognized the baseline images significantly better than the distractor images and exhibited a significant and selective deficit in the recognition of target-paired images (compared to young-adult healthy controls). These results confirm an age-related impairment in the temporal selective attention processes at the basis of the ABE and further support the hypothesis that healthy ageing implicates an increase in the attentional resources required by the detection task, which in turn offsets the attentional facilitation enjoyed by target-paired stimuli [39]. Notably, our data indicate that this impairment is not limited to older participants between 60 and 75 years (as in Bechi Gabrielli et al. [39]); rather, when the study-test delay is sufficiently long, a sizable decrease in the magnitude of the ABE can already be observed in participants between 36 and 60 years.

A number of hypotheses can be put forward regarding the cerebral mechanisms that underlie the reduction of the positive effects of the ABE in healthy adult controls and bipolar patients. Currently, the neural underpinnings of the ABE are poorly understood [9]. An fMRI study by Swallow, Makovski and Jiang [49] reported that the regions that responded more strongly to target than distractor stimuli comprised those typically activated in attentional selection tasks, including the anterior insula, the anterior cingulate, the intraparietal sulcus, the supramarginal gyrus, the precuneus, the basal ganglia, and the posterior brain stem in the vicinity of the locus coeruleus. Similarly, Bechi Gabrielli et al. [50] found that, compared to the processing of distractor-associated stimuli, the encoding of target-associated images produced a greater activation of regions within the ventral frontoparietal network, including the temporoparietal junction, the supramarginal area, the anterior cingulate cortex, and several subcortical regions. Interestingly, some of these areas were found to be dysfunctional in previous fMRI studies examining the performance of bipolar patients in sustained attention tasks. For example, Diwadkar et al. [51] showed that an increase in the attention demands of the detection task led to increased engagement of the frontal-striatal pathway in healthy controls, but disengagement in adolescents with a higher genetic risk for bipolar disorder. The already mentioned study by Sepede et al. [36] reported that, during errors in target detection, both patients and relatives showed a larger activation in the bilateral insula and the posterior part of the middle cingulate cortex. Finally, Brooks, Bearden, Hoblyn, Woodard, and Ketter [52] found that the omission errors of euthymic bipolar patients were strongly related to dorsolateral prefrontal hypometabolism and greater paralimbic, insula, and cingulate hypermetabolism. Although additional studies are needed to clarify the neural bases of the ABE and the differences between healthy and clinical populations, it seems reasonable to hypothesize that the reduction of the ABE in bipolar patients might be ascribed to a dysfunction of the ventral frontoparietal network.

In this respect, it should be highlighted that a significant deficit in the recognition of target-paired stimuli (coupled with an intact recognition of distractor-paired stimuli) has now been reported in a growing number of studies investigating the ABE in several psychiatric diseases, including amnestic mild cognitive impairment, post-traumatic stress disorder, schizophrenia, and bipolar disorder [18,19,20,21]. Most interestingly, from a clinical standpoint, two recent meta-analyses have pointed out that hypoactivation in brain regions regulating the ABE might signal vulnerability to develop different forms of psychopathology. For example, McTegue et al. [53] showed that, in tasks of cognitive control, hypoactivation in the right inferior prefrontal/insular cortex represented a transdiagnostic feature of schizophrenia, bipolar disorder, major depressive disorder, anxiety disorders, and substance use. Similarly, Janiri et al. [54] found that, in mood disorders, post-traumatic stress disorder, and anxiety disorders, the most consistent transdiagnostic abnormalities in task-related brain activity were identified in the inferior prefrontal cortex/insula, the inferior parietal lobule, and the putamen. Clearly, then, interventions aimed at improving the patients’ performance in the ABE paradigm, targeting at least part of these shared brain phenotypes, might also improve clinical outcomes and reduce or prevent morbidity in the general population (see Kèri et al. [18] for an example).

From a clinical point of view, the present results may be relevant for translational neuroscience and psychiatry, especially with regards to the role of the hippocampus in the formation of bound representations linking the background stimuli with the central target items. A previous study by Szamosi and colleagues [20] reported that the hippocampal volume was positively associated with the recognition of target-paired images in the ABE paradigm, for both older controls and patients with amnestic mild cognitive impairment. Moreover, significant shrinkage of the hippocampal formation has been reported for older adults [42], as well as in several psychiatric populations, including individuals with bipolar disorder [55] and schizophrenic patients [56]. If, as suggested by the evidence described above, the ABE paradigm shows sensitivity to hippocampal pathology, then the recognition of target-paired images might be successfully used to detect the early stages of a wide range of clinical memory disorders [20].

The present study has some limitations that must be taken into account. First, all euthymic bipolar patients were under pharmacological treatment, usually with mood-stabilizing and antipsychotic treatments [57,58], and this might have influenced their neurocognitive performance. However, data from literature show that the attentional deficits of these patients endure after controlling for mild residual symptomatology [29] and pharmacological treatment [30,31]. We are therefore inclined to believe that the significant impairment in the recognition of target-paired stimuli was genuine. Second, we used a relatively long interval between the encoding phase and the recognition task (20 min). Since Prull [17] found that healthy older adults exhibited an intact ABE when tested after 2 min from the encoding phase, investigating whether a significant ABE can be observed in bipolar patients after a short study-test interval represents an important avenue for future research. 

## 5. Conclusions

In conclusion, our results are consistent with previous evidence showing attentional deficits in bipolar patients during the remission phase of the disease. In this clinical population, the absence of the ABE was mediated by a specific difficulty in the recognition of target-paired images, suggesting that temporal selective attention processes are defective in bipolar patients [4]. Based on the idea that the ABE represents a trade-off between attentional boost and attentional competition, we propose that the maintenance of a fast and accurate performance in the detection task is more attentionally demanding for patients than for healthy controls and that the increase in the negative effects of attentional competition is enough to eliminate the ABE. Our second important result is the absence of ABE in healthy adult controls. This confirms and extends the conclusions reported by Bechi Gabrielli et al. [39] and provides further evidence that the boosting mechanisms associated with target detection undergo an age-related decrease starting from about 35 years. Future studies should clarify the cerebral mechanisms leading to early attenuation of the ABE in healthy adults.

## Figures and Tables

**Table 1:** Mean scores for the demographic and cognitive measures of euthymic bipolar patients (BP) and healthy control subjects (HC) in the two age subgroups (young-adults and adults). Standard errors are reported in parentheses. For the WAIS-IV subtests, weighted scores are reported.

Variables	Young-Adults		Adults	
	BP (*n* = 12)	HC (*n* = 15)	BP (*n* = 30)	HC (*n* = 27)
Age (years)	27.4 (1.8)	26.7 (1.6)	50.3 (1.1)	51.1 (1.2)
Education (years)	13.7 (1.0)	15.5 (0.9)	12.7 (0.6)	13.5 (0.7)
Gender (M/F)	7/5	9/6	13/17	11/16
Digit Span (forward)	8.7 (0.8)	9.5 (0.7)	8.7 (0.5)	9.7 (0.5)
Digit Span (backward)	8.5 (0.9)	10.1 (0.8)	8.1 (0.6)	9.7 (0.6)
Symbol Search	8.9 (0.7) ^a^	10.9 (0.7) ^b^	8.4 (0.5) ^a^	10.5 (0.5) ^b^
Digit Symbol-Coding	10.2 (0.7)	12.1 (0.6)	8.3 (0.4) ^a^	10.2 (0.7) ^b^

Note. The superscripts a and b indicate significant differences (*p* < 0.05) between couples of BP and HC means.

## Data Availability

Data available on request. The data presented in this study are available on request from the corresponding author.
